# Modulation of the growth and metabolic response of cyanobacteria by the multifaceted activity of naringenin

**DOI:** 10.1371/journal.pone.0177631

**Published:** 2017-05-12

**Authors:** Beata Żyszka, Mirosław Anioł, Jacek Lipok

**Affiliations:** 1 Department of Analytical and Ecological Chemistry, Faculty of Chemistry, University of Opole, Opole, Poland; 2 Department of Chemistry, Faculty of Food Science, Wrocław University of Environmental and Life Sciences, Wrocław, Poland; Texas A&M University College Station, UNITED STATES

## Abstract

The interactions between the plant-derived bioflavonoid, naringenin, and prokaryotic microalgae representatives (cyanobacteria), were investigated with respect to its influence on the growth and metabolic response of these microorganisms. To achieve reliable results, the growth of cyanobacteria was determined based on measurements of chlorophyll content, morphological changes were assessed through microscopic observations, and the chemical response of cells was determined using liquid and gas chromatography (HPLC; GC-FID). The results show that micromolar levels of naringenin stimulated the growth of cyanobacteria. Increased growth was observed for halophilic strains at naringenin concentrations below 40 mg L^-1^, and in freshwater strains at concentrations below 20 mg L^-1^. The most remarkable stimulation was observed for the freshwater species *Nostoc muscorum*, which had a growth rate that was up to 60% higher than in the control. When naringenin was examined at concentrations above 40 mg L^-1^, the growth of the tested microorganisms was inhibited. Simultaneously, an intensive excretion of exopolysaccharides was observed. Microscopic observations strongly suggest that these effects resulted from a structural disturbance of cyanobacterial cell walls that was exerted by naringenin. This phenomenon, in combination with the absorption of naringenin into cell wall structures, influenced cell permeability and thus the growth of bacteria. Fortunately, almost all the naringenin added to the culture was incorporated into to cell substructures and could be recovered through extraction, raising the possibility that this modulator could be recycled.

## Introduction

A growing interest in using cyanobacteria in biotechnological processes is reflected in the increasing number of published papers and patents. Many biotransformations are currently carried out using plant-derived substances as substrates. However, little information is available concerning the interactions of these photosynthetic bacteria with natural compounds of plant origin, such as flavonoids, which are released into aquatic environments. Described examples are related to the presence of flavonoids in cyanobacterial cells and the culture media used to grow them [1–5], with cells being reported to accumulate these natural compounds to near millimolar levels when grown in different media [6]. In addition to the influence of these compounds on gene expression in cyanobacteria [7,8], the ability of plants that contain flavonoids to inhibit the growth of this microalgae was also observed [9–11]. Nevertheless, the interactions between natural compounds of plant origin and cyanobacteria remain poorly understood, especially with regards to the unknown mechanism(s) of metabolic responses of these microorganisms. Thus, the study of such an interaction is a reasonable approach.

Naringenin (4’,5,7-trihydroxyflavanone) is a citrus bioflavonoid that is classified as a flavanone and exerts multiple biological effects. As natural compound, naringenin is primarily obtained from the pericarp and pulp of *Rutaceae* fruits, such as grapefruits, tangerines and oranges. It is also the major effective ingredient of some herbs used in Chinese medicine, such as *Drynaria fortunei*, *Fructus aurantii immaturus*, *Fructus aurantii seu poncirus* and *Exocarpium citri rubrum* [12]. It possesses numerous therapeutic properties that include antibacterial, antifungal, antihepatotoxic, antioxidant, antispasmodic, antiulcer, antiestrogenic and anticancer activities. Naringenin is also known to indirectly modulate the metabolism of many xenobiotics [13]. Therefore, we initially studied this substrate with the expectation it would be transformed by cyanobacteria, generating new derivatives with interesting properties. Instead, we found that this compound affects the growth and metabolic response of these microalgae.

Flavonoids act in vitro as effective antimicrobials against a wide array of microorganisms. Plant phenolic compounds can interfere with the growth of microorganisms, but they can act also as synergists to increase the effectiveness of preservatives. It is thought that low concentrations of phenolic compounds affect enzymes connected to ATP formation in cells, and at high concentrations, they induce protein aggregation [14]. These substances can also modify the permeability of bacterial cell membranes by altering the composition of fatty acids and phospholipid head-group in the membrane or interacting with membrane proteins [15, 16]. Although the exact mechanisms are unknown, flavonoids inhibit some important cellular functions in bacteria, including nucleic acid synthesis [17], the functioning of the cytoplasmic membrane [18] and energy metabolism [19]. These effects are thought to be the result of metabolic perturbation due to enzyme inhibition [19]. It has been reported that in bacterial cells, transition metal ions of metalloenzymes, such as phosphatases, can form strong ligand complexes with flavonoids [20]. In addition, ion channels, which are sensitive components of prokaryotic cells, are especially susceptible targets of flavonoids action [19]. Flavonoids are also known to induce changes in the molecular weight, structure, and level of secretion of some microbial extracellular proteins, exopolysaccharides (EPS) and lipopolysaccharides (LPS) [21].

EPS are metabolic products that are comprised of polysaccharides, proteins, nucleic acids, phospholipids and humic acids that accumulate on the surface of microbial cells and provide protection against the harsh external conditions by stabilizing the structure of the membrane [22]. A previous study found that supplementing media with naringenin had no significant effect on the growth and protein content of *Rhizobium meliloti* cells, though it increased exopolysaccharide production [23, 24]. Another study showed that the presence of naringenin induced the synthesis of some extracellular proteins and suppressed the production of EPS in a strain of *Sinorhizobium fredii* [21], which shares a common cellular structure with cyanobacteria.

Cyanobacteria (blue-green algae) comprise a unique phylum of gram-negative bacteria that are the only known prokaryotes capable of oxygen-evolving photosynthesis. These microorganisms occupy diverse ecological niches and exhibit enormous diversity in terms of their habitats, physiology and metabolic capabilities. Cyanobacteria appear able to address many types of stress, including chemical stresses. Information regarding the activity of flavonoids towards cyanobacteria is scarce, but relevant literature does suggest that flavonoids could have significant effects on the growth and physiological functions of cyanobacteria. The most important seem to be the disruption of the photosynthetic system, the compromision (deterioration) of membrane integrity, induction of membrane depolarization and permeabilization of cells [11].

Our study was focused on the effects the citrus derived flavonoid naringenin has on representative halophilic and freshwater cyanobacteria. The impact of naringenin on cell growth, morphology, and excretion of extracellular compounds, as well as the fate of naringenin within cells, is presented and discussed with respect to their beneficial attributes.

## Materials and methods

### Chemicals

All media components were purchased from POCH S.A. (Avantor Performance Materials Poland S.A., Gliwice, Poland). All chemicals used were of analytical grade. Naringenin (98% pure) was purchased from Sigma-Aldrich (W530098) (Poznan, Poland). Naringenin stock solutions (1.5, 3.0, 6.0, 12.0, 18.0, 24.0 and 42.0 mg mL^-1^) were prepared in dimethyl sulfoxide (DMSO) and were sterilized by filtration prior being added to the medium. Stock solutions were prepared the day of use and were stored in the dark to prevent photodamage. To assure that equivalent amounts of DMSO were added to all the cultures that received naringenin, all naringenin stock solutions were prepared using DMSO (Table 1). A total of 100 μL of each stock solution was added to the corresponding 30 mL of medium to ensure all cultures contained equivalent amounts of DMSO (0.33%, v/v), which did not have a toxic effect on cyanobacterial cells and was not responsible for the reported cellular effects.

**Table 1:** Concentrations of naringenin in stock solutions and experimental media.

	Concentration of naringenin						
**Stock solution in DMSO [mg mL**^**-1**^**]**	1.5	3.0	6.0	12.0	18.0	24.0	42.0
**Experimental media [mg L**^**-1**^**]**	5	10	20	40	60	80	100

### Strains and culture conditions

The cyanobacteria used in our experiments that occur naturally in hypersaline ponds included *Spirulina platensis* [strain C1 (PCC9438)], *Arthrospira maxima* [strain CCALA 027], *Arthrospira fusiformis* [strain CCALA 023], while those from freshwater ponds, lakes and streams that were used included *Anabaena sp*. [strain CCALA 007], *Anabaena laxa* [strain CCALA 805], *Nodularia moravica* [strain CCALA 797], *Chroococcus minutus* [strain CCALA 055] and *Nostoc cf*. *muscorum* [strain CCALA 129]. An axenic culture of *Spirulina platensis* was obtained from the Pasteur Culture Collection (PCC) (Institut Pasteur, Paris), whereas all other cyanobacterial species were purchased from the Culture Collection of Autotrophic Organisms (CCALA) (Institute of Botany AS CR, Trebon, Czech Republic).

All tested microorganisms were grown in standard media to obtain seed inoculums (MSp (American Type Culture Collection (ATCC) 1679) or BG11 (ATCC 616) media for hypersaline or freshwater strains, respectively), which were then used to initiate the experimental cultures. Cyanobacteria were subcultured every three weeks by transferring 10 mL aliquots to 50 mL of fresh MSp, or BG11 medium, depending on the strain [25]. All tested microorganisms were grown at 24±1°C, with a 16 h day (approx. 1000 lx light intensity) and 8 h night photoperiod, in 250-mL Erlenmeyer flasks containing 60 mL of cultures. Similar conditions were maintained to support the growth of experimental cultures.

### Experimental cultures

The experimental cyanobacteria cultures were initiated by transferring appropriate volumes of 21-day-old exponential phase subcultures into fresh media. The volumes of inocula used were established experimentally by determining the concentration of chlorophyll, which was 1 mg L^-1^ for all the tested species at the beginning of culturing. Microorganisms were cultured for 14 days in the appropriate standard media supplemented with appropriate naringenin stock solutions that resulted in final concentrations of naringenin of 5, 10, 20, 40, 60, 80, or 100 mg L^-1^ (Table 1). Sterile media containing an appropriate amount of naringenin, but without cyanobacterial cells acted as a substrate controls to determine substrate stability in the medium, whereas appropriate sterile media inoculated with the examined bacteria, but without naringenin, were used as culture controls. Cultures for each experiment, including the controls, were carried out at least in triplicate.

### Determination of the growth of microorganisms

The growth of the examined photoautotrophs was assessed by time-course measurements of total chlorophyll content in experimental cultures. At 3–4 day intervals, three 1.0 mL samples were taken from each experimental or control replicate culture, and the cells were sedimented by centrifugation at 13.000 x g for 5 min. The cell pellets were resuspended in 0.9 mL of methanol, and chlorophyll solubilization was allowed to proceed for 20 min in the dark, with occasional mixing. Samples were then centrifuged as before, and the total chlorophyll content in the supernatant was determined spectrophotometrically using Arnon’s formula (total chlorophyll (a+b) = 20.21 A_645_+8.02 A_663_) [26], using a Hitachi U 2810 spectrophotometer (Hitachi High-Technologies Europe GmbH, Krefeld, Germany). Growth curves were generated by plotting the average levels of chlorophyll in each experimental or control culture replicate. The growth rates of the examined photoautotrophs were calculated from the slopes of obtained growth curves. Finally, the ratio of the growth rates of experimental cultures with respect to the appropriate controls was determined to illustrate the differences in the cyanobacterial growth dynamics and allow for comparisons between experiments. The percentage values (including standard deviations) of the ratios of growth rates were correlated with the appropriate concentration of naringenin, tabularized and are presented in the results. Additionally, the growth of the tested cyanobacteria was examined by determination of the dry mass at the beginning and end of the experiments. To do this, cultures were filtered through previously dried and weighed microbial filters; then, the filters were dried and weighed until the difference between successive measurements was not higher than 5%.

The significance of the observed differences among samples was assessed using common statistical analyses. All data are presented as the means ± standard deviation (SD). Statistical differences from the control were determined by Student t-test using significance levels at p < 0.0001 (****), p < 0.001 (***), p < 0.01 (**) and p < 0.05 (*). The confidence limits were additionally verified according to Snedecor and Cochran [27] using Statistica software package Version 7.1 (StatSoft).

### Examination of the effect of naringenin on cyanobacterial cell morphology

#### Observations of cyanobacterial cell morphology using optical microscopy

Changes in the microscopic cell morphology of the cultured microorganisms were observed using an Olympus CX41 optical microscope (Olympus Europe, Hamburg, Germany) at 100x, 400x or 1000x magnification and were imaged with a dedicated Olympus camera.

#### Microscopic analysis of cyanobacterial cells by scanning electron microscopy (SEM)

SEM was used to examine the surface topology and distribution of specimens at a high magnification [28]. The cells from control and experimental cultures were pelleted by centrifugation at 13.000 x g for 5 min and were fixed with 3% glutaraldehyde buffered with 0.2 M phosphate buffer at room temperature for 24 h. The specimens were washed three times with 0.1 M phosphate buffer (pH = 7.2), post-fixed with 1% osmium tetroxide in 0.1 M phosphate buffer for 4 h at room temperature in a light-tight container, and then were washed three times with 0.1 M phosphate buffer. The samples were then dehydrated by successively soaking them in increasing concentrations of ethanol (50 to 70% in 10% increments). Dehydrated specimens were treated with an ion-spotter-coating of gold in a vacuum evaporator and subjected to an SEM analysis using a HITACHI TM-3000 TableTop Scanning Electron Microscope (Hitachi High-Technologies Europe GmbH, Krefeld, Germany).

### Assessment of the fate of naringenin in cyanobacterial cultures

Cells were separated from culture medium by filtration followed by centrifugation at 5.000 x g for 1 min. Qualitative and quantitative determinations of naringenin in culture media were made from 30 mL volumes of each medium, which were extracted two times with 15 mL of ethyl acetate, after which both extracts were combined, dried over anhydrous magnesium sulphate and the solvent was evaporated to dryness using a vacuum evaporator. The remaining residues were dissolved in 1 mL of methanol and subjected to planar chromatography (TLC), UV-Vis spectrophotometry, gas chromatography (GC-FID) and high-performance liquid chromatography (HPLC) analyses.

The accumulation of naringenin in cyanobacterial cells was assessed by determining the presence of this compound in the biomass extracts. To do this, cyanobacterial cells were harvested at the end of experiments by centrifugation, immediately freeze-dried and were then stored at -20°C before being lyophilized. Before extraction, lyophilised cells were sonicated for 30 min in ethyl acetate (3 mL) using ultrasonic bath (40 kHz, 100 W; Branson, Danbury, Ct, USA), and for a further 30 min using a Hielscher UP200HT ultrasonic homogenizer (26 kHz, 200 W, Hielscher Ultrasonics GmbH, Teltow, Germany). Resulting homogenates were centrifuged at 5.000 x g for 1 min, and the supernatants were pooled and concentrated to 1 mL. The amount of naringenin was determined by TLC and gas chromatography with flame ionization detection (GC-FID) comparing sample retention times with those of a naringenin standard.

#### UV-Vis measurements of culture media

Continuous spectra of samples were recorded with a Hitachi U 2810 spectrophotometer (Hitachi High-Technologies Europe GmbH, Krefeld, Germany) in quartz cuvettes with an optical pathway of 1 cm. Scans were recorded from 250 nm to 400 nm at room temperature (23–25°C) at a speed of 200 nm/min, paying particular attention to the analytical wavelengths of flavanones (280 and 330 nm) [29].

#### HPLC analysis of culture media

The changes in the chemical composition of the experimental and control culture media were assessed by reversed phase high-performance liquid chromatography (HPLC) using a Dionex Ultimate 3000 HPLC system (Thermo Fischer Scientific, Waltham, Massachusetts, USA) equipped with diode array detector (DAD-3000RS). The mobile phase consisted of two solvents: A—MeCN; B—1% HCOOH in H_2_O. A 28 min gradient elution was used with the following conditions: 1 min 50/50%, A/B; 15 min 50 to 100% A; 5 min 100% A; 2 min 100 to 50% A; 5 min 50/50% A/B. The samples were filtered through non-sterile syringe filters with a pore size of 0.45 μm. A total of 20 μl of each prepared sample was injected onto a C-18 Hypersil Gold column (150 x 4.6, Thermo Scientific Part N 25005–154630), with the retention times and peak intensities of separated compounds assessed at 290 and 360 nm.

#### Thin layer chromatographic separation of the cell biomass extracts

TLC was carried out using silica gel plates (DC Kieselgel 60/TLC Silica gel 60, Merck) using chloroform:methanol (10:1) as the eluent. The plates were sprayed with a solution containing 10 g of Ce(SO_4_)_2_ and 20 g of phosphomolybdic acid in 1 L of 10% H_2_SO_4_ to visualize the presence of flavonoids, which give yellow to brown-orange coloured spots. After spraying, the plates were gently heated until coloured spots appeared. The retention coefficients of the spots were determined and compared with that of a naringenin standard.

#### GC-FID analysis of the extracts from cyanobacterial cells

Chromatographic separation in gas phase was performed using a Thermo Scientific FOCUS GC equipped with a Flame Ionization Detector (Thermo Fischer Scientific, Waltham, Massachusetts, USA) and a HP-5 fused silica capillary column (30.0 m x 0.25 mm i.d., film thickness 0.25 μm). The programmed temperature was 100–275°C at 10°C min^-1^ with 1.0 min hold at 100°C and 17 min hold at 275°C. The injector temperature was set to 250°C. The flow rate of the carrier gas (helium) was set at 1.5 mL min^-1^. A split flow of 30 mL min^-1^ and a split ratio of 20:1 was used. The chromatographic data were recorded and processed using the OpenChrom software [30].

## Results and discussion

### The influence of naringenin on the growth of cyanobacteria

**Fig 1:**
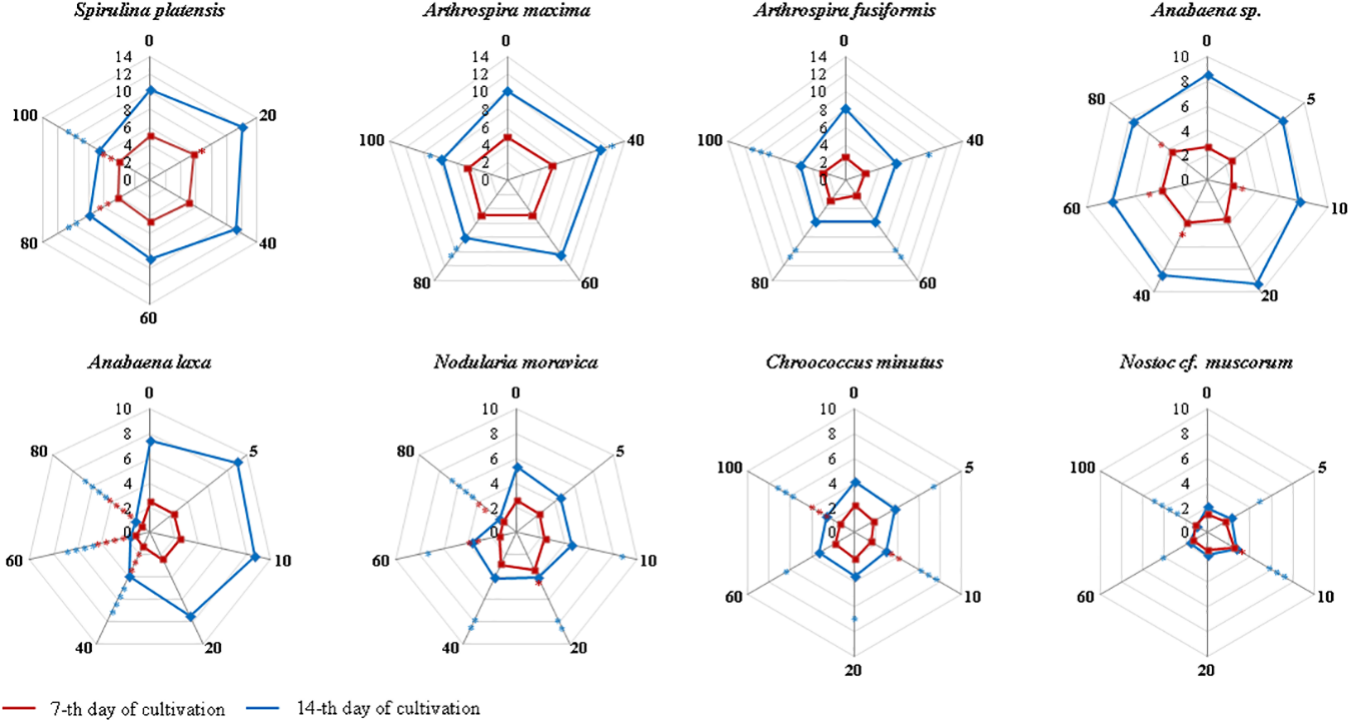
Interdependence of the total chlorophyll content (μg mL^-1^) in day 7 (red) and day 14 (blue) cultures of halophilic and freshwater cyanobacteria and naringenin concentration, with the naringenin concentrations indicated on the appropriate radical axes (spider web graphs). The axes indicated as “0” belong to the appropriate controls (cultures without naringenin). The points that indicate the contents of chlorophyll on the axes that indicate various concentrations of naringenin reflect a stimulatory effect when they are above the relevant value on axis “0”, whereas the points located below this value, reflect an inhibitory action. All values are the means from at least five independent experiments (n = 5). The significance of the influence of naringenin on chlorophyll content is shown in the form of asterisks (*p < 0.05; **p < 0.01; ***p < 0.001; ****p < 0.0001) was tested against appropriate controls.

The sensitivity of individual species of cyanobacteria to different concentrations of naringenin is illustrated in Fig 1. The data for the 7th and 14th days of cultivation reflected the response of the cultures that from the beginning of experiment were influenced by naringenin in various concentrations. The resulted “images” after 7 and 14 days of cultivation, reflects the sensitivity of cells, which developed as suppressed colony that possibly had a different metabolic response.

The total chlorophyll content after appropriate days of culturing halophilic and freshwater species of blue green algae indicated that naringenin, even at the highest tested concentration, only had a limited effect on the growth of the tested cyanobacteria, with exception of *Anabaena laxa*, *Nodularia moravica* and *Nostoc cf*. *muscorum*, which did not exhibit cell mortality.

The growth of *Anabaena laxa* and *Nodularia moravica* was significantly inhibited in media that contained 60 or 80 mg L^-1^ naringenin, whereas *Nostoc cf*. *muscorum* was only inhibited in the presence of 100 mg L^-1^ of naringenin. It is worth noting that in the latter case the amount of chlorophyll in the culture was stable over the course of the experiment and that the cells of this strain remained alive for two weeks afterwards. This finding suggests that the generational replacement, in which the dead cells were replaced by emerging ones, was the reason for the observed phenomenon. From an analysis of the microbial growth rates, it can be concluded that two halophilic strains (*Spirulina platensis* and *Arthrospira maxima*), and one freshwater species (*Anabaena* sp.) were resistant to naringenin whereas one halophile (*Arthrospira fusiformis*) and three freshwater species (*Chroococcus minutus*, *Nodularia moravica*, and *Nostoc cf*. *muscorum*) were sensitive (Table 2).

**Table 2:** The ratios of the growth rates of experimental cultures with respect to the growth rate of appropriate controls at certain concentrations of naringenin, expressed as percentage values ± s.d.

	Microorganism	The ratio of the growth rates of experimental cultures with respect to the growth rate of appropriate controls [%]						
		5	10	20	40	60	80	100
**Halophilic cyanobacteria**	*Spirulina platensis*	(-)	(-)	119±5	110±11	81±11	69±10	57±3
	*Arthrospira maxima*	(-)	(-)	(-)	112±8	104±6	73±3	69±7
	*Arthrospira fusiformis*	(-)	(-)	(-)	75±10	70±10	67±2	62±3
**Freshwater cyanobacteria**	*Anabaena* sp.	88±13	84±10	108±9	93±3	86±6	81±8	(-)
	*Anabaena laxa*	125±1	116±1	102±1	55±3	11±1	7±1	(-)
	*Nodularia moravica*	(-)	(-)	77±5	66±8	59±8	25±1	(-)
	*Chroococcus minutus*	95±1	68±1	76±8	(-)	70±8	(-)	54±5
	*Nostoc* cf. *muscorum*	123±1	167±6	83±19	(-)	59±10	(-)	-2±17

Naringenin effectively stimulated the growth of freshwater *Nostoc* cf. *muscorum* by approximately 20 and 60% at concentrations of 5 and 10 mg L^-1^ of naringenin, respectively. In the case of *Anabaena laxa*, the same concentrations of naringenin caused a 25 and 16% stimulation of growth, respectively. The stimulatory effect was also observed for the halophilic species *Spirulina platensis* and *Arthrospira maxima* and did not exceed 20% when naringenin concentrations lower than 40 mg L^-1^ or 60 mg L^-1^ were respectively used. Nevertheless, the stimulatory effect, which influenced the proliferation and growth of the valuable nutraceutical strain *Spirulina platensis*, seemed to almost be a “ready to use factor” that increased the productivity of this cyanobacterium.

It may be stated, therefore, that for the majority of the studied microorganisms, the higher the concentration of naringenin used in the growth medium, the higher the inhibition of cyanobacterial growth was as determined by chlorophyll content. Similar conclusions were drawn based on the measurements of cyanobacterial biomass. The average weight of the appropriate experimental cultures confirmed, among other factors, the beneficial effect of lower concentrations of naringenin on the growth of *Spirulina platensis* and *Arthrospira maxima*, which had 120–125% improved growth in relation to the respective controls. Moreover, the results of these measurements indicated that in each experiment, the biomass of the culture was adequate to determine the chlorophyll content.

The inhibitory effects of flavonoids towards the growth of microorganisms vary depending on the type and size of flavonoid architecture and the positions of its side chains in the backbone [31]. The inhibitory activity exerted by naringenin is typical for flavonoids, whereas the stimulation of growth may have resulted from the existence of appropriate metabolic attributes, since some microbial strains, including cyanobacteria, may contain low amounts of phenolic compounds, such as flavonoids [2]. However, some natural flavones were observed to cause significant inhibition of the growth of the freshwater cyanobacterium *Microcystis aeruginosa* [11]. Nevertheless, the results obtained here suggest that flavonoids, represented in this study by naringenin, could have significant effects on the growth of cyanobacterial cultures and that this effect occurs in a dose dependant manner.

### The effect of naringenin on the integrity of cyanobacterial cells

To verify how and to what extent, naringenin influenced the integrity of cyanobacterial cells, light microscopic analyses at magnifications up to 1000x were carried out. The highest tested concentrations of naringenin provoked some damage to the integrity of the bacterial cell walls, especially in the freshwater strains. Representative microphotographs of the cells of freshwater cyanobacteria growing in media supplemented with naringenin clearly exhibit a much higher secretion of some extracellular compounds in relation to the appropriate control. These exudates accumulated close to the surface of the microbial cells. It is worth emphasizing that freshwater species typically generate large amounts of such substances than do halophilic strains. Moreover, the higher the concentration of naringenin used, the greater the amount of exudate was observed.

The structure of the exudates, after bearing in mind their presumable polysaccharide character, was verified using ^1^H NMR. According to the literature, ^1^H NMR spectra of polysaccharides can be divided into two regions: those with chemical shifts ranging from 3.2 to 4.4 ppm, which correspond to sugar ring protons, and those with a chemical shift in the range of 4.4 to 5.6 ppm, related to anomeric protons [32]. The ^1^H NMR spectra of the coloured substances excreted by cyanobacteria contained sugar ring signals in the chemical shift region of 3.2 to 4.4 ppm, and the well separated peaks in the region corresponding to the anomeric protons at 5.15 ppm were characteristic for released polysaccharides (RPS) and at approximately 5.1 ppm were characteristic for capsular exopolysaccharides (CPS). These results confirmed the exopolysaccharide character of the substances released from the cyanobacterial cells by the action of naringenin. Since only a few species of bacteria are known for the production of EPS at high levels, the possibility of identifying new producers (under applied naringenin stress) is of importance as a possible commercial source of these compounds [33].

### Scanning electron microscopy (SEM) analysis of the morphology of cyanobacterial cells affected by naringenin

**Fig 2:**
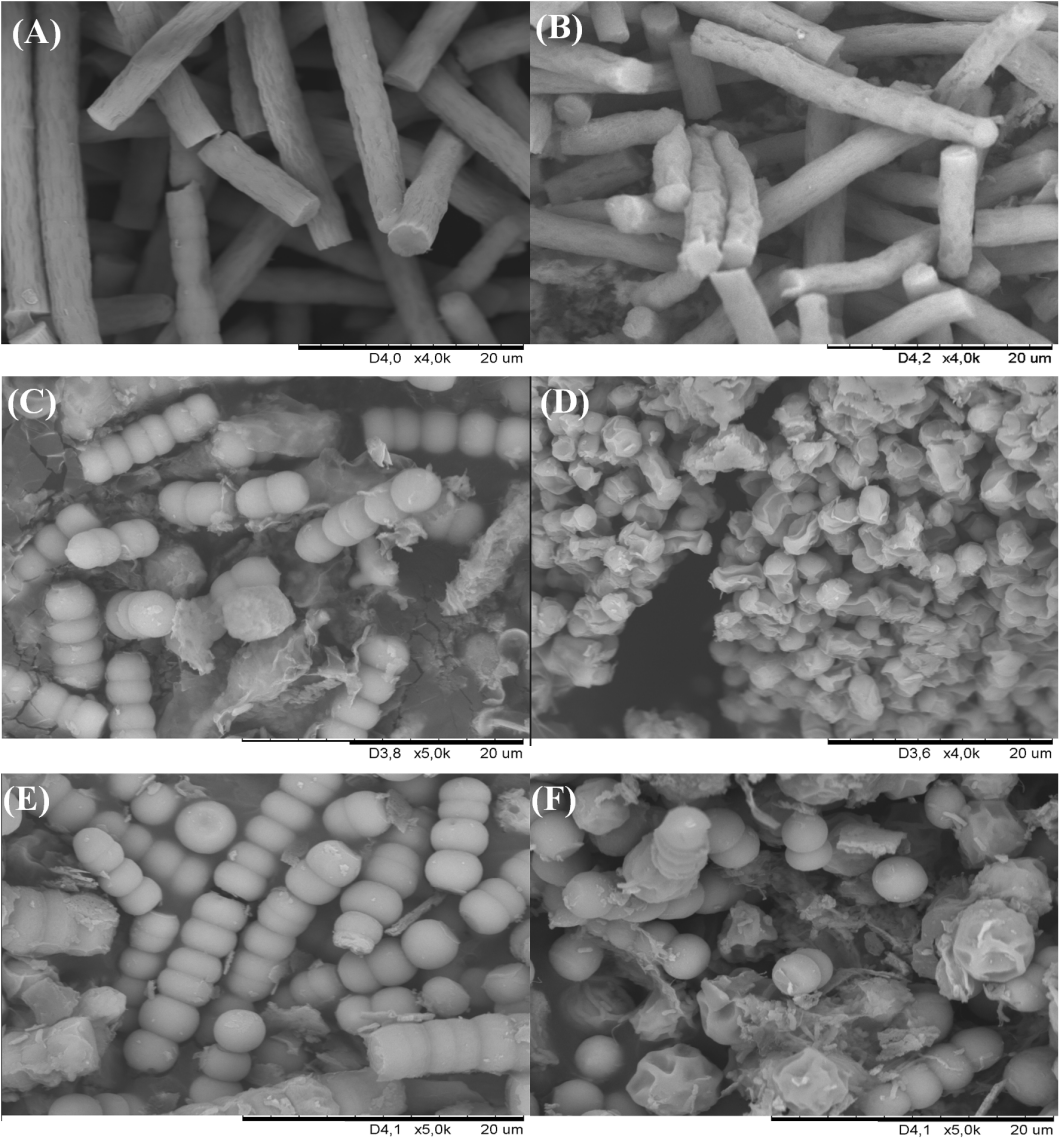
The representative SEM photographs of *Spirulina platensis* (A), *Spirulina platensis* + N (naringenin) (100 mg L^-1^) (B), *Anabaena laxa* (C), *Anabaena laxa* + N (80 mg L^-1^) (D), *Nodularia moravica* (E), *Nodularia moravica* + N (80 mg L^-1^) (F) illustrate the differences in the structure of the cyanobacterial cell walls between the control and experimental cultures.

The exposure of the cyanobacterial strains to naringenin resulted in morphological changes in the structure of the cell walls, as shown in Fig 2A–2F.

Cyanobacterial cells from the control cultures had regular shapes and sleek surfaces, whereas the cells incubated with naringenin possessed irregular shapes and rough surfaces. The exposure of cyanobacteria to naringenin caused visible collapses in cell surface topology and produced wrinkled abnormalities with numerous small clefts, which were regularly distributed on the surface of the cyanobacterial cells. This effect was observed for all the tested cyanobacterial species. Such morphological features of bacterial cells may be caused by the lysis of the outer membrane, followed by the loss of electron-dense materials on the surface. The loss of electron dense material from the cells treated with naringenin indicates a loss of cell constituents and a breakdown of the cell walls, resulting in the facilitated release of inner-cell material [34,35]. Morphological analyses of freshwater cyanobacteria treated with naringenin exhibited higher amounts exopolysaccharides than were observed in the halophilic strains. This effect, however, is not easily seen on SEM microphotographs because to demonstrate some morphological changes in the structure of cyanobacterial cell walls, the parts of the surface not covered by the aforementioned exudates were subjected to SEM analysis. Similar morphological alterations were observed for gram-negative bacteria such as *Staphylococcus aureus*, *Helicobacter pylori* [36] and *Escherichia coli* [37].

It is also known that natural flavones compromised the membrane integrity and induced membrane depolarization and permeabilization in cells of *Microcystis aeruginosa* [11].

The above results suggest that flavonoids could have meaningful effects on physiological functions in cyanobacterial species.

### The fate of naringenin in cyanobacterial cultures

#### The presence of naringenin in liquid media of cyanobacterial cultures

**Fig 3:**
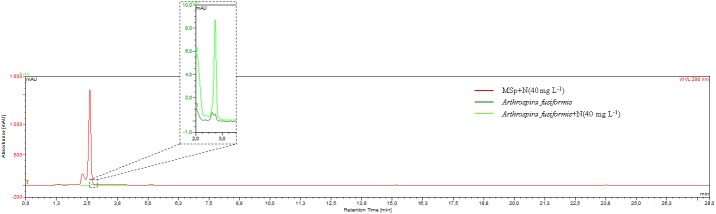
The representative superimposed HPLC chromatograms of the substrate control containing 20 mg L^-1^ of naringenin in MSp or BG11 media and the experimental media of *Arthrospira fusiformis*, obtained at a wavelength of 290 nm. The magnification window contains part of the superimposed chromatogram, which presents the measured intensity of the peak of naringenin detected in postharvest medium of *A*. *fusiformis*.

Eight studied cyanobacterial strains were screened for their ability to biotransform naringenin. HPLC analyses indicated that even at the highest tested concentrations of naringenin (80 and 100 mg L^-1^), none of examined microorganisms transformed this flavonoid into any new metabolite(s) of polyphenolic character. Simply put, no peaks reflecting the presence of analytes of phenolic character were observed in any of the chromatograms. Even more surprising, after fourteen days of the growth of cyanobacteria, the concentration of naringenin in the supernatants of all experimental cultures decreased at least 160-fold to trace levels. The intensities of the naringenin peaks ranged from 2 to 10 mAU (Fig 3), whereas in the substrate control, a well-separated peak of naringenin had an intensity of approximately 1600 mAU. This indicated the disappearance of the flavonoid from the medium. A representative chromatogram is shown in Fig 3, but similar observations were made for the additional strains of cyanobacteria tested.

### The presence of naringenin in cell biomasses

The low concentration of naringenin in two-week old growth media could be the result of metabolization of this substance or possibility from naringenin being bound to the cytoplasmic membrane of cells. A strong affinity for similar structures, as cyanobacterial cell-walls have a pH of 5.7 or higher, was previously mentioned in the literature [21]. The pH-dependent hydrophobicity of naringenin is reflected by the changes observed in its UV spectrum, thus, the spectrum at pH 5.5 shows a single absorption maximum at a wavelength of 289 nm, which is correlated inversely to the peak at approximately 320 nm at pH 10.5. This phenomenon suggests that the existence of at least two pH-dependent forms of naringenin exist that possess different electronic properties [33]. Therefore, the thesis that the absorption of naringenin by cells was caused through its accumulation into the cytoplasmic membranes seems reasonable and explains the observed changes in morphology of the cell surfaces.

**Fig 4:**
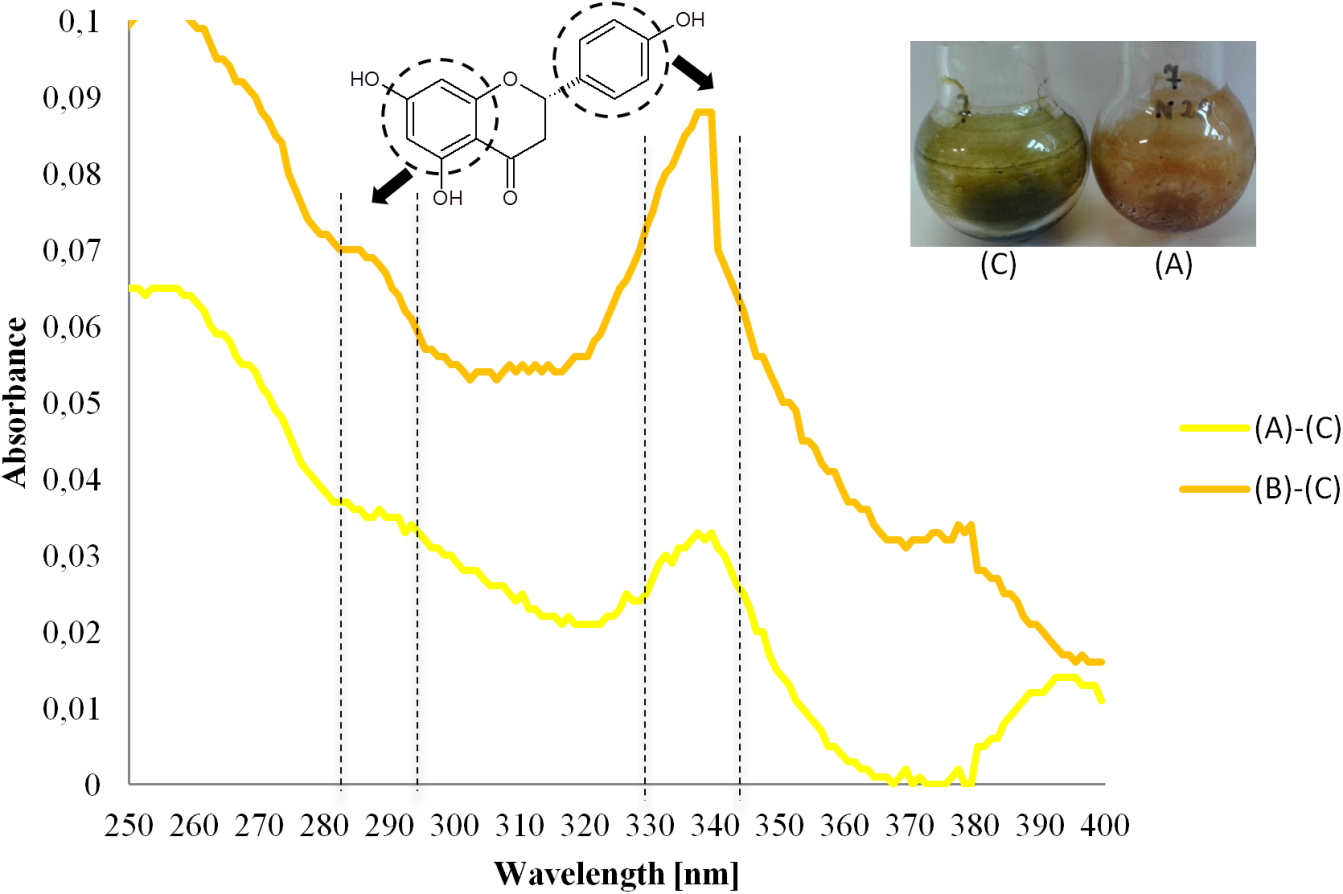
A representative figure of different colours of the extracts of *Anabaena* sp. cell extracts: green-grey extract from cell biomass of control (C) and orange-green of the extract of the experimental culture supplemented with 20 mg L^-1^ of naringenin (A). The presence of two maxima (ca. 290 and ca. 330 nm) of absorption in the UV-VIS differential (“experiment” *vs*. “control”) spectra of the extracts from the cell biomass of *Anabaena* sp. treated with (A) 20 mg L^-1^ and (B) 60 mg L^-1^ of naringenin revealed the presence of this flavanone.

To verify this hypothesis, extractions of the cell biomasses with ethyl acetate were carried out. The comparison of the colours of the concentrated extracts from the control cells (green-grey) with those obtained from the experimental cells, which had grown in the presence of at least 20 mg of naringenin (orange-green), indicated a significant difference in their chemical composition (Fig 4). To check if this difference was the direct effect of the presence of naringenin, UV-VIS measurements supplemented with TLC and GC-FID chromatographic analysis were performed.

The data obtained in UV-VIS measurements were matched and mathematically transformed to form the differential spectra. In this attempt, the results of subtraction “experiment–control” comprise of two maxima at approximately 290 and 330 nm that are characteristic for a more hydrophobic form of naringenin. The results of the TLC analysis conclusively revealed that naringenin was present in the extracts from the cells. The R_f_ values of relative spots visualized using cerium phosphomolybdate, were nearly the same as the retention factor of the naringenin standard. Interestingly, the interpretation of the location and character of the detected spots led to the observation that cyanobacteria natively produce polyphenolic compounds other than naringenin. This result is consistent with the literature, since some cyanobacterial strains were found to contain low amounts of phenolic compounds, including flavonoids [2].

**Fig 5:**
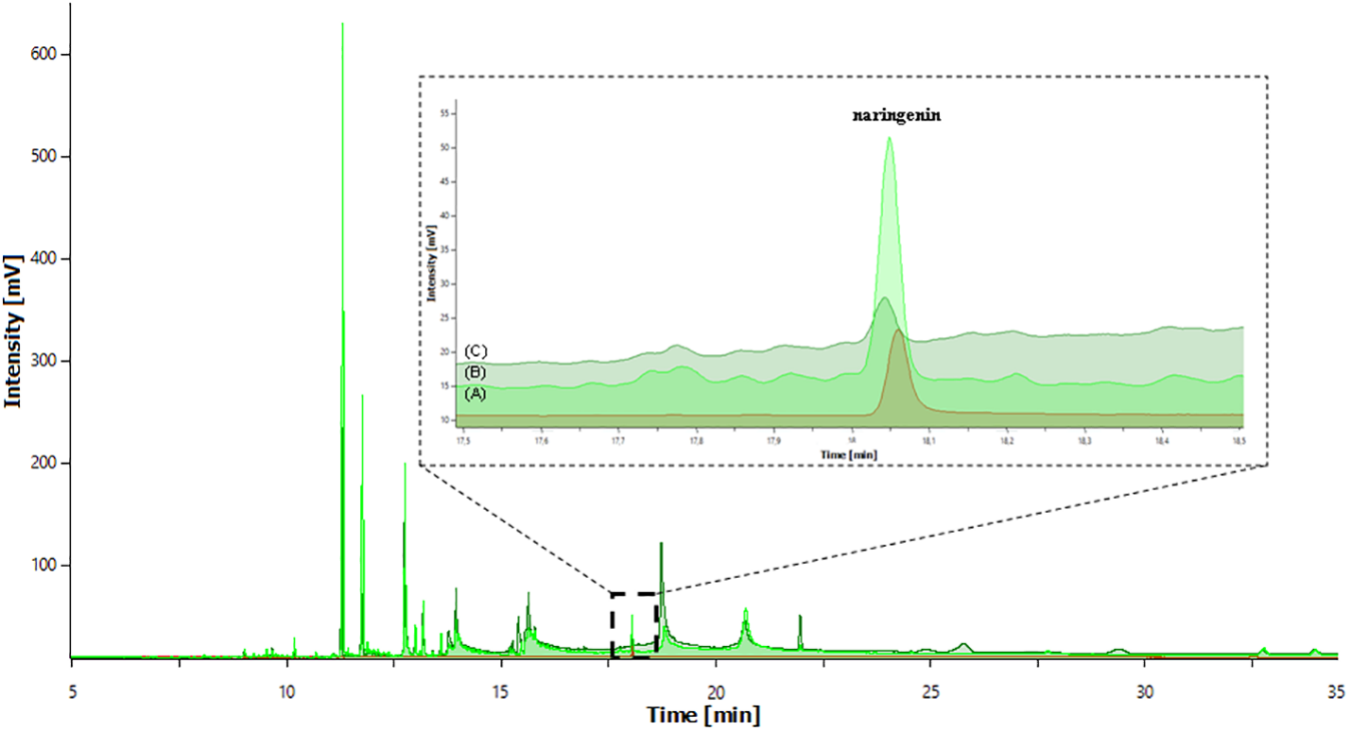
The representative GC-FID superimposed chromatograms of the extracts from the cells of *Anabaena* sp. and from the substrate control. The precise location and intensity of the peaks of the naringenin standard (A), natively produced undefined polyphenolic compound (C) and the substances extracted from the culture supplemented with naringenin (B) are present in the magnification window.

Similar results were obtained during GC-FID analyses of the extracts from the biomasses of experimental cultures and the undoubted presence of naringenin was shown. Moreover, the peak of a polyphenolic substance, detected in planar chromatography as natively produced by *Anabaena* sp., was also recorded. The value of the retention time of this peak was 18.04 min, whereas the retention time of naringenin is 18.06 min (Fig 5).

What should be emphasized is that the areas of relevant peaks recorded in extracts from experimental cultures supplemented with 20 mg L^-1^ of naringenin (average area 1336 ± 88) versus the control culture (exhibiting only natively produced flavonoids of peak area of 254 ± 22) and versus the peak of naringenin recorded from a 10 mg L^-1^ solution (peak area of 527 ± 31) (Fig 5) indicated not only the presence of naringenin but also its almost 100% recovery from the biomass. Similar results were obtained for other strains,; therefore, only a representative chromatogram is presented. This suggested that naringenin can be bound to the cell wall structures of cyanobacteria without modification of their chemical nature; thus, it can be effectively recovered afterwards in the whole range of tested concentrations.

## Conclusions

Naringenin beneficially influenced the growth of cyanobacteria by changing the permeability of the cell walls and cellular membranes in a manner that depended on its concentration, and on the strain. This phenomenon was accompanied with the absorption of naringenin into cell wall structures, and thus might result in a leakage of intracellular material, e.g., intensive exudation of EPS. Therefore, naringenin influences the growth and physiological functions of halophilic and fresh-water cyanobacteria. The passage of almost the entire amount of naringenin into the cells, where this flavanone was merged into cell substructures (mostly membranes), creates the unique opportunity to obtain the fraction of excreted exopolysaccharides, free from the flavanone responsible for its facilitated release. Fortunately, almost the entire amount of naringenin, which was merged into cell substructures, might be recovered through the extraction, creating an opportunity to recycle this modulator. These features of this natural substance are of special concern, since any modification of microbial metabolism, which is not involved with the use of genetic engineering and can have certain benefits, is highly desirable. Moreover, if such a modification results from the impact of a natural compound, it is more easily accepted by society.

## References

[1] Scholz B, Liebezeit G. Chemical screening for bioactive substances in culture media of microalgae and cyanobacteria from marine and brackish water habitats: First results. Pharm Biol. 2006;44: 544–549.

[2] Singh DP, Prabha R, Meena KK, Sharma L, Sharma AK. Induced accumulation of polyphenolics and flavonoids in cyanobacteria under salt stress protects organisms through enhanced antioxidant activity. Am J Plant Sci. 2014;5: 726–735.

[3] Goiris K, Muylaert K, Voorspoels S, Noten B, De Paepe D, Baart GJE, et al Detection of flavonoids in microalgae from different evolutionary lineages. J Phycol. 2014;50: 483–492. doi: 10.1111/jpy.12180 26988321 10.1111/jpy.12180

[4] Babić O, Kovač D, Rašeta M, Šibul F, Svirčev Z, Simeunović J. Evaluation of antioxidant activity and phenolic profile of filamentous terrestrial cyanobacterial strains isolated from forest ecosystem. J Appl Phycol. 2016; 28: 2333–2342.

[5] Hossain MF, Ratnayake RR, Meerajini K, Wasantha Kumara KL. Antioxidant properties in some selected cyanobacteria isolated from fresh water bodies of Sri Lanka. Food Sci Nutr. 2016; 4: 753–758. doi: 10.1002/fsn3.340 27625779 10.1002/fsn3.340 PMC5011383

[6] Sharathchandra K, Rajashekhar M. Antioxidant activity in the four species of cyanobacteria isolated from a sulfur spring in the Western Ghats of Karnataka. Int J Pharm Bio Sci. 2013;4: 275–285.

[7] Cohen MF, Yamasaki H. Flavonoid-induced expression of a symbiosis-related gene in the cyanobacterium *Nostoc punctiforme*. J Bacteriol. 2000;182: 4644–4646. 10913102 10.1128/jb.182.16.4644-4646.2000 PMC94640

[8] Cohen MF, Sakihama Y, Takagi YC, Ichiba T, Yamasaki H. Synergistic effect of deoxyanthocyanins from symbiotic fern *Azolla* spp. on hrmA gene induction in the cyanobacterium *Nostoc punctiforme*. Mol Plant Microbe In. 2002;15: 875–882. 10.1094/MPMI.2002.15.9.875 12236594

[9] Yan R, Wu Y, Yang L, Fang Y, Kerr PG. *Scutellaria baicalensis* georgi controls cyanobacterial blooms and benefits to aquatic ecosystem. Fresen Environ Bull. 2011;20: 18–25.

[10] Ahmad A, Kim SH, Ali M, Park I, Kim JS, Kim EH, et al New chemical constituents from oryza sativa straw and their algicidal activities against blue-green algae. J Agr Food Chem. 2013; 61: 8039–8048. 23889328 10.1021/jf402145u

[11] Huang H, Xiao X, Ghadouani A, Wu J, Nie Z, Peng C, et al Effects of natural flavonoids on photosynthetic activity and cell integrity in *Microcystis aeruginosa*. Toxins. 2015;7: 66–80. doi: 10.3390/toxins7010066 25584428 10.3390/toxins7010066 PMC4303814

[12] Wang MJ, Chao PDL, Hou YC, Hsiu SL, Wen KC, Tsai SY. Pharmacokinetics and conjugation metabolism of naringin and naringenin in rats after single dose and multiple dose administrations. J Food Drug Anal. 2006;14: 247–253.

[13] Bugianesi R, Catasta G., Spigno P, D’Uva A, Maiani G. Naringenin from cooked tomato paste is bioavailable in men. J Nutr. 2002;132: 3349–3352. 12421849 10.1093/jn/132.11.3349

[14] Nychas GJE. Natural antimicrobials from plants In: Gould GW, editors. New methods of food preservation. London: Blackie Academic & Professionals; 1995 pp. 58–89.

[15] Ultee A, Kets EPW, Alberda M, Hoekstra FA, Smid EJ. Adaptation of the food-borne pathogen *Bacillus cereus* to carvacrol. Arch Microbiol. 2000a;174: 233–238. 11081791 10.1007/s002030000199

[16] Poklar Ulrih N, Ota A, Šentjurc M, Kure S, Abram V. Flavonoids and cell membrane fluidity. Food Chem. 2010;121: 78–84.

[17] Plaper A, Golob M, Hafner I, Oblak M, Solmajer T, Jerala R. Characterization of quercetin binding site on DNA gyrase. Biochem Biophys Res Commun. 2003;306: 530–536. 12804597 10.1016/s0006-291x(03)01006-4

[18] Mirzoeva OK, Grishanin RN, Calder PC. Antimicrobial action of propolis and some of its components: the effects on growth, membrane potential and motality of bacteria. Microbiol Res. 1997;152: 239–246. doi: 10.1016/S0944-5013(97)80034-1 9352659 10.1016/S0944-5013(97)80034-1

[19] Cushnie TPT, Lamb AJ. Antimicrobial activity of flavonoids. Int J Antimicrob Ag. 2005;26: 343–356. 10.1016/j.ijantimicag.2005.09.002 PMC7127073 16323269

[20] Ahmad A, Kaleem M, Ahmed Z, Shafiq H. Therapeutic potential of flavonoids and their mechanism of action against microbial and viral infections–A review. Food Res Int. 2015;77: 221–235.

[21] Lin CC, Chen YC, Song SC, Lin LP. Flavonoids as inducers of extracellular proteins and exopolysacchrides of *Sinorhizobium fredii*. Biol Fertil Soils. 1999;30: 83–89.

[22] McSwain BS, Irvine RL, Hausner M, Wilderer PA. Composition and distribution of extracellular polymeric substances in aerobic flocs and granular sludge. Appl Environ Microbiol. 2005;7: 1051–1057. 10.1128/AEM.71.2.1051-1057.2005 PMC546788 15691965

[23] Jain V, Nainawatee HS. Flavonoids influence growth and saccharide metabolism of *Rhizobium meliloti*. Folia Microbiol. 1999;44: 311–316.

[24] Recourt K, van Brussel AA, Driessen AJ, Lugtenberg BJ. Accumulation of a nod gene inducer, the flavonoid naringenin, in the cytoplasmic membrane of *Rhizobium leguminosarum* biovar viciae is caused by the pH-dependent hydrophobicity of naringenin. J Bacteriol. 1989;17: 4370–4377. 10.1128/jb.171.8.4370-4377.1989 PMC210214 2753859

[25] Forlani G, Prearo V, Wieczorek D, Kafarski P, Lipok J. Phosphonate degradation by *Spirulina* strains: Cyanobacterial biofilters for the removal of anticorrosive polyphosphonates from wastewater. Enzyme Microb Tech. 2011;48: 299–305. 10.1016/j.enzmictec.2010.12.005 22112915

[26] Poora RJ. The chequered history of the development and use of simultaneous equations for the accurate determination of chlorophylls a and b. Photosynth Res. 2002;73: 149–56. doi: 10.1023/A:1020470224740 16245116 10.1023/A:1020470224740

[27] Snedecor GW, Cochran WG. Statistical Methods, eighth ed., Iowa State University Press Ames, Iowa; 1989.

[28] Goldstein JI, Newbury DE, Echlin P, Joy DC, Fiori C, Lifshin E. Scanning Electron Microscopy and X-Ray Microanalysis. Plenum Press: New York; 1981.

[29] Andersen OM, Markham KR. Flavonoids: Chemistry, Biochemistry and Applications. CRC Press/Taylor & Francis: Boca Raton; 2006

[30] Wenig P, Odermatt J. OpenChrom: a cross-platform open source software for the mass spectrometric analysis of chromatographic data. BMC Bioinformatics. 2010;11: 405 doi: 10.1186/1471-2105-11-405 20673335 10.1186/1471-2105-11-405 PMC2920884

[31] Moon SH, Lee JH, Kim KT, Park YS, Nah SY, Ahn DU, Paik HD. Antimicrobial effect of 7-O-butylnaringenin, a novel flavonoid, and various natural flavonoids against *Helicobacter pylori* strains. Int J Environ Res Public Health. 2013;10: 5459–5469. doi: 10.3390/ijerph10115459 24169409 10.3390/ijerph10115459 PMC3863854

[32] Sheng GP, Yu HQ. Characterization of extracellular polymeric substances of aerobic and anaerobic sludge using three-dimensional excitation and emission matrix fluorescence spectroscopy. Water Res. 2006;40: 1233–1239. doi: 10.1016/j.watres.2006.01.023 16513156 10.1016/j.watres.2006.01.023

[33] Wang Y, McNeil B. Production of the fungal exopolysaccharide scleroglucan by cultivation of *Sclerotium glucanicum* in airlift reactor with an external loop. J Chem Tech Biotechnol. 1995;63: 215–222.

[34] Shin SY, Bajpai VK, Kim HR, Kang SC. Antibacterial activity of eicosapentaenoic acid (EPA) against foodborne and food spoilage microorganisms. LWT—Food Sci Technol. 2007;40: 1515–1519.

[35] Becker-Ritt AB, Martinelli AH, Mitidieri S, Feder V, Wassermann GE, Santi L, et al Antifungal activity of plant and bacterial ureases. Toxicon. 2007;50: 971–983. doi: 10.1016/j.toxicon.2007.07.008 17825863 10.1016/j.toxicon.2007.07.008

[36] Martini S, D’Addario C, Colacevich A, Focardi S, Borghini F, Santucci A, et al Antimicrobial activity against *Helicobacter pylori* strains and antioxidant properties of blackberry leaves *(Rubus ulmifolius)* and isolated compounds. Int J Antimicrob Ag. 2009;34: 50–59. 10.1016/j.ijantimicag.2009.01.010 19386474

[37] Lee KA, Moon SH, Kim KT, Mendonca AF, Paik HD. Antimicrobial effects of various flavonoids on *Escherichia coli* O157:H7 cell growth and lipopolysaccharide production. Food Sci Biotechnol. 2010;19: 257–261.

